# A comparative modeling study on non-climatic and climatic risk assessment on Asian Tiger Mosquito (*Aedes albopictus*)

**DOI:** 10.7717/peerj.4474

**Published:** 2018-03-19

**Authors:** Farzin Shabani, Mahyat Shafapour Tehrany, Samaneh Solhjouy-fard, Lalit Kumar

**Affiliations:** 1 School of Environmental and Rural Science, University of New England, Armidale, NSW, Australia; 2 Geospatial Science, College of Science, Royal Melbourne Institute of Technology, Melbourne, Australia; 3 School of Environmental and Rural Science, University of New England, Australia

**Keywords:** Mosquito, Ae. albopicus and *Aedes albopictus*, MaxEnt, USA, GIS, Evidential Belief Function

## Abstract

*Aedes albopictus*, the Asian Tiger Mosquito, vector of Chikungunya, Dengue Fever and Zika viruses, has proven its hardy adaptability in expansion from its natural Asian, forest edge, tree hole habitat on the back of international trade transportation, re-establishing in temperate urban surrounds, in a range of water receptacles and semi-enclosures of organic matter. Conventional aerial spray mosquito vector controls focus on wetland and stagnant water expanses, proven to miss the protected hollows and crevices favoured by *Ae. albopictus.* New control or eradication strategies are thus essential, particular in light of potential expansions in the southeastern and eastern USA. Successful regional vector control strategies require risk level analysis. Should strategies prioritize regions with non-climatic or climatic suitability parameters for *Ae. albopictus*? Our study used current *Ae. albopictus* distribution data to develop two independent models: (i) regions with suitable non-climatic factors, and (ii) regions with suitable climate for *Ae. albopictus* in southeastern USA. Non-climatic model processing used Evidential Belief Function (EBF), together with six geographical conditioning factors (raster data layers), to establish the probability index. Validation of the analysis results was estimated with area under the curve (AUC) using *Ae. albopictus* presence data. Climatic modeling was based on two General Circulation Models (GCMs), *Miroc3.2* and *CSIRO-MK30* running the RCP 8.5 scenario in MaxEnt software. EBF non-climatic model results achieved a 0.70 prediction rate and 0.73 success rate, confirming suitability of the study site regions for *Ae. albopictus* establishment. The climatic model results showed the best-fit model comprised Coldest Quarter Mean Temp, Precipitation of Wettest Quarter and Driest Quarter Precipitation factors with mean AUC value of 0.86. Both GCMs showed that the whole study site is highly suitable and will remain suitable climatically, according to the prediction for 2055, for *Ae. albopictus* expansion.

## Introduction

Invasive alien species pose a threat to biodiversity, ecosystems, agriculture, human and animal health, and consequently inflict economic damage (Pyšek & Richardson, 2010). Invasive weeds smother and crowd out indigenous flora, thereby threatening local fauna; some release allergenic pollens harmful to many humans, while invasive waterweeds clog and choke natural waterways. The introduction of an alien flora species may concurrently introduce alien parasites, fungi, invertebrate larvae or diapausing eggs, hosted by that species in its environmental niche. Such hosted species may be potential vectors of novel pathogens into their new environment. Similarly, some invasive alien insect species may be vectors of diseases of epidemic potential (Antia et al., 2003) that can be medically, socially and economically devastating (Pimentel, 2011). Despite the advanced control mechanisms of modern public health, and stringent standards imposed at borders to control what travelers and traders carry in and out through border posts, invasive alien species still penetrate and establish an environmental presence. Whether or not the potential health and economic impacts of such invasions have been quantified, logic demands the elimination of such potentially dangerous invasive alien species as a precaution, as quickly as possible (Wittenberg & Cock, 2001). In practice, aside from invasions of pests that have an economic impact and act as vectors of disease, response is often delayed (Hulme, 2006).

*Aedes albopictus*, or the Asian tiger mosquito, a belligerent insect that bites during the day, has emerged as a threat to public health worldwide and has been identified as the vector of the Chikungunya and Dengue viruses, among others. Most recently it has been verified in Brazil that *Ae. albopictus* is a potential vector of Zika virus, of which its closest relative, *Ae. Aegypti,* has been the major vector thus far (Schaffner, Medlock & Bortel, 2013). *Ae. albopictus* is one of the world’s one hundred worst invasive species according to the Global Invasive Species Database (GISD, 2017). This devastating impact has been facilitated by a rapid spread from its native East Asian to western Pacific and Indian Ocean natural domains (Caminade et al., 2012).

While the species has had multiple introductions to Australia and New Zealand, it has not established itself there, mainly attributable to the efficiency of entomological surveillance in the airports and harbors of these countries (Ritchie et al., 2006).

*Ae. albopictus* was established in the USA in 1980, ostensibly arriving in a shipload of used tires from Japan. (Rai, 1991). Once *Ae. albopictus* establishes in a particular locality, eradication becomes virtually impossible, and costly vigilance and control becomes essential (Holder et al., 2010).

The observed suitable climate for *Ae. albopictus* growth now ranges from temperate through sub-tropical to tropical, with vegetation from savanna to evergreen and Amazon forest. It can adapt to both arid and humid conditions (Kraemer et al., 2015a; Messina et al., 2016; Vega-Rúa et al., 2014). Winter temperatures appear to be a limiting factor of further spreading of the species (Hanson & Craig, 1994; Rochlin et al., 2013a; Thomas et al., 2012), while winter precipitation may moderate the suitability of the species to colder temperatures (Hanson & Craig, 1995). The natural *Ae. albopictus* habitat was originally forest edges where they bred in tree holes, stumps of bamboo and bromeliads. Thus, the species was formerly classified as a specifically rural vector (Higa, 2011). However, *Ae. albopictus* has demonstrated an exceptional ability to adapt to new environmental conditions and establish itself. In urban and suburban environments, it may be found breeding in manmade containers such as external water tanks, animal water troughs, bird baths, plant containers, moist organic matter and abandoned tires, in both towns and suburbs (Caputo et al., 2012). The species is now considered the major vector, and in certain areas the sole vector, of such environments (Caputo et al., 2012). Invasive alien mosquitos often displace the indigenous mosquito territorially. However, the only known case of an invasive alien replacing another invasive alien mosquito species is the displacement of *Ae. Aegypti* by *Ae. albopictus* (Kenis et al., 2009), which has been researched and corroborated in Florida, USA. *Ae. Aegypti* is the major international vector of Zika, and *Ae. albopictus* has been recently acknowledged as a potential vector, which will certainly have an impact on an outbreak of Zika in any region of such displacement (Ding et al., 2018; Kenis et al., 2009; Nihei et al., 2014; Simard et al., 2005; Weaver & Lecuit, 2015). It should be mentioned that Ding et al. (2018) have recently mapped the spatial distribution of *Aedes aegypti* and *Aedes albopictus* for the current time through temperature suitability, NDVI, precipitation, urban accessibility, night time light, urban regions, relative humidity, and population density.

USA public health departments in both rural and urban communities, which previously had no need for developing strategies to control mosquitos, now face the challenge of *Ae. albopictus* (Rochlin et al., 2013a), which poses a threat to the region without the development of novel methods of control. Existing mosquito controls generally have involved aerial spraying of easily accessible marshland and floodwater breeding grounds. However, the *Ae. albopictus* partiality for smaller scale, protected breeding in close range of humans in water storage and other moist semi-enclosed artifacts, evade existing control methods. The alternative means the necessity for communities falling within the paths of expansion to deal with the impact. The involvement of a complete community is vital. The crucial issue is whether new strategies can be developed at a relatively low cost. Projecting regions of expansion and general forward planning and sufficient funding through greater public awareness will be the key to effective campaigns. In terms of *Ae. albopictus* adaptation from rural to urban surrounds, and the need for policy makers and public health organizations to prioritize resources, crucial decisions will need to be made on whether to focus generally on regions with suitable climate, or rather on specific non-climatic parameters such as roads, lakes, rivers, altitude and slope within climatically suitable regions?

The answer to this question is synonymous with the aim of this study which sets out to determine whether *Ae. albopictus* distribution is more associated with non-climatic parameters or climate suitability?

The current distribution of *Ae. albopictus* in the southeastern region of the USA was used to develop two separate models for this species of mosquito, based on (i) regions with suitable non-climatic factors, and (ii) regions with suitable climate. The non-climatic model comprised a data-driven Evidential Belief Function with six conditioning factors: (i) altitude, (ii) slope, (iii) aspect, (iv) distance of locality from road, (v) distance of locality from river and (vi) geology, through ArcGIS. The climatic model was based on two GCMs of *Miroc3.2* and *CSIRO-MK30* for the current time under RCP 8.5 scenario and employed MaxEnt software. We hold the view that the methodology and results of this study will promote active surveillance of *Ae. albopictus*, as well as other invasive insect species. The results of this study will emphasize the need for increasing awareness to promote vigilance and effective control and eradication mechanisms, complementing the current online information networks of the relevant government and non-government bodies.

## Methodology

### Study area selection

**Figure 1:**
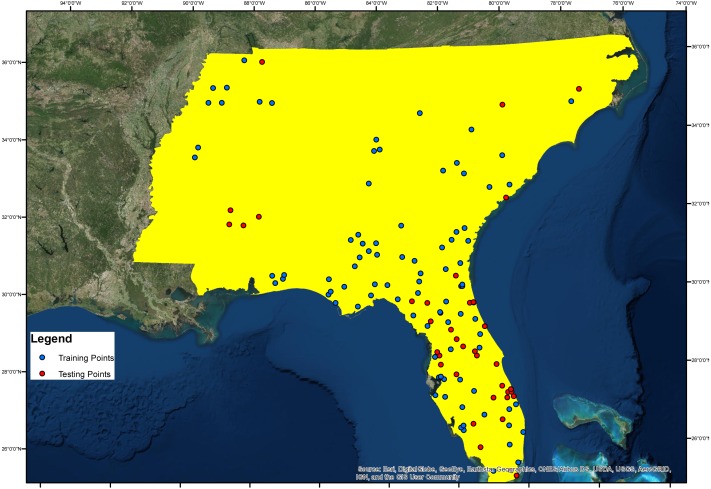
Study area and Asian Tiger Mosquito testing and training points.

The study site is located between 75°30′00″W and 92°00′00″W, and 25°00′00″N and 36°30′00″N in USA (Fig. 1). In selecting the study site, we looked for an area exhibiting variations of each conditioning factor, as well as *Ae. albopictus* presence. For example, in terms of altitude, the study area should display a range of altitudes. Our selected study area had an altitude range from 0 m to 2,031 m above sea level. For geology, the study area had 80 different geological fractures.

### Spatial datasets

#### Inventory factors

In the study, 70% of the *Ae. albopictus* presence layer, an inventory factor obtained from Global Biodiversity Information Facility database (Global Biodiversity Information Facility, 2017) and Kraemer et al. (2015b), were used for model training while the remainder 30% was reserved for model validation (Fig. 1). The training and testing points cover all the study area and the testing points were selected randomly.

#### Conditioning factors

**Figure 2:**
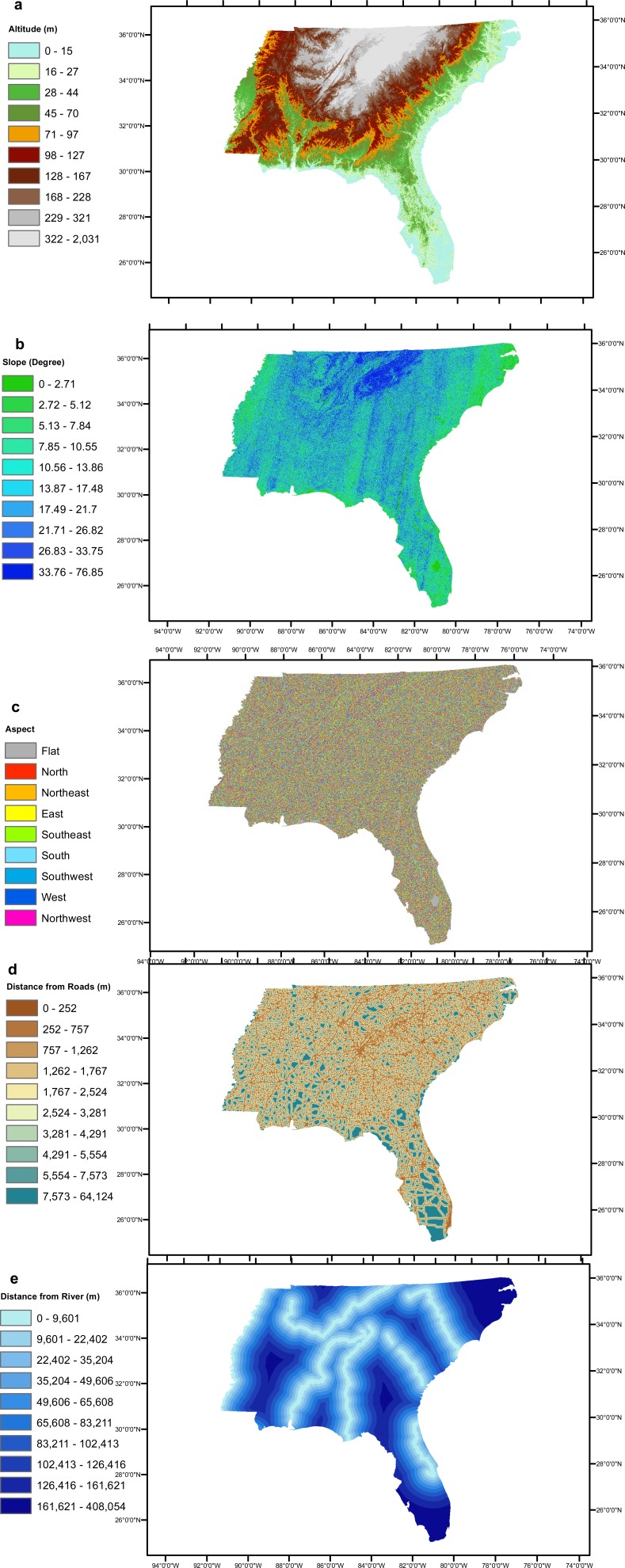
Study area’s (A) altitude (B) slope, (C) aspect, (D) distance from roads, (E) distance from rivers.

**Figure 3:**
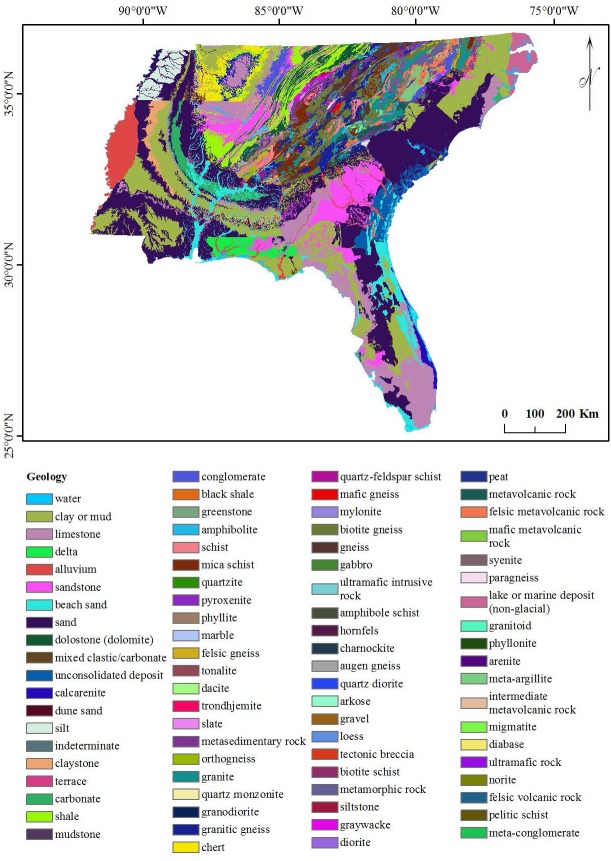
Geology of the study area.

The six geographical conditioning factors (i) altitude, (ii) slope, (iii) aspect, (iv) distance of locality from road, (v) distance of locality from river and (vi) geology, with a grid cell size 90 × 90 m were used to run the EBF model. The quantile classification scheme was used for all conditioning factors, as recommended by Tehrany, Pradhan & Jebur (2013). Altitude, slope and aspect layers were generated from DEM data obtained from EarthExplorer (2014) as shown in Figs. 2A–2C respectively. Distances from road and river layers were generated by Euclidean Distance tool and divided into ten classes using the quantile method, as shown in Figs. 2D and 2E respectively. The geology layer, obtained from the United States Department of Agriculture (USDA) (United States Department of Agriculture, 2017) contained 80 different types of lithology as shown in Fig. 3. The elevation layer was included as it depicts climate variations and the physical barriers limiting dispersion. The road, river and geological layers were included as the greatest densities of *Ae. albopictus* occur in urban environments (Rochlin et al., 2013a).

### Non-climatic modeling

Evidential Belief Function (EBF), which is also called Dempster-Shafer theory of evidence, was developed by Dempster (Dempster, 1967), based on the Bayesian theory of subjective probability. Its advantages are the relative flexibility with which it accepts uncertainty and its ability to aggregate beliefs from many sources of evidence (Thiam, 2005). Rather than estimating the validity of probabilities, the Dempster-Shafer technique calculates the nearness of the evidence in proving the validity of a hypothesis (Pearl, 1990). Applications of EBF have been effective in many fields of research that utilize GIS data (Malpica, Alonso & Sanz, 2007).

To produce a hazard index of presence of *Ae. albopictus*, the conditioning factors were expressed individually as acquired weights and then aggregated (Eq. (1)).

Assuming a set of *Ae. albopictus* presence conditioning factors *C* = (*C*_*i*_, *i* = 1, 2, 3, …, *n*), consisting of mutually exclusive and exhaustive factors *C*_*i*_. *C* represents the frame of discernment, and a fundamental probability assignment is represented by the function *m*:*P*(*C*) → [0, 1].

The set *P*(*C*) includes all subsets of *C*, as well as *C* itself and the empty set. *m*:*P*(*C*) → [0, 1] is described as a mass function and satisfies *m*(Φ) = 0 and ∑_*AC*_*m*(*A*) = 1, in which Φ represents the empty set and *A* represents any subset of *C*. The *m*(*A*) estimates to what degree the evidence supports *A*, which is denoted by *Bel* (*A*), a belief function.

There are four basic evidential belief functions attributable to a proposition, based on evidence. These four functions establish the degree of: (i) Belief (*Bel*), (ii) Disbelief (*Dis*), (iii) Uncertainty (*Unc*) and (iv) Plausibility (*Pls*). *Bel* represents the lower bound and *Pls* represents the upper bound of probability (Althuwaynee, Pradhan & Lee, 2012; Awasthi & Chauhan, 2011). *Unc* is established by the difference between *Bel* and *Pls*, and represents the ignorance. *Dis* represents the degree of probability that the proposition is false.

*Dis* = 1 − *Pls* or 1 − *Unc* − *Bel*, such that *Bel* + *Unc* + *Dis* = 1. For a case of *C*_*ij*_ zero presence of *Ae. albopictus*, implying that *Bel* = 0, *Dis* is reset to zero, whether that is the case or not (Carranza, Hale & Faassen, 2008). EBF can be estimated on the basis of a subjective judgment or calculated on the input of data (Srivastava, Mock & Gao, 2011). By superimposing the inventory map (L) of *Ae. albopictus* onto the six individual conditioning factor maps, we ascertained the number of pixels with *Ae. albopictus* presence and absence, for each separate conditioning factor. Assuming that *N*(*L*) represents the total of presence pixels and *N*(*C*) the total pixels comprising the study site, *C*_*ij*_ represents the *j*th class attribute of *Ae. albopictus* presence conditioning factors *C*_*i*_ ( *i* = 1, 2, …, *n*), *N*(*C*_*ij*_) is the total of pixels for class *C*_*ij*_, and *N*(*L*∩*C*_*ij*_) is the *Ae. albopictus* presence pixels in *C*_*ij*_. According to (Carranza & Hale, 2003), estimation of EBFs based on data is represented by:

(1) \begin{eqnarray*}& & Bel \left( {C}_{ij} \right) = \frac{{W}_{{C}_{ij}(Ae.\;Albopictus\;\text{presence})}}{\sum _{j=1}^{n}{W}_{{C}_{ij}(Ae.Albopictus\text{absence})}} \end{eqnarray*}

(2) \begin{eqnarray*}& & {W}_{{C}_{ij}(Ae.\;Albopictus\;\text{presence})}= \frac{ \frac{N(L\cap {C}_{ij})}{N(L)} }{ \frac{[N({C}_{{}_{ij}})-N(L\cap {C}_{ij})]}{[N(C)-N(L)]} } \end{eqnarray*}

(3) \begin{eqnarray*}& & Dis \left( {C}_{ij} \right) = \frac{{W}_{{C}_{ij}(Ae.\;Albopictus\;\text{absence})}}{\sum _{j=1}^{n}{W}_{{C}_{ij}(Ae.\;Albopictus\;\text{absence})}} \end{eqnarray*}

where,

(4) \begin{eqnarray*}{W}_{{C}_{ij}(Ae.\;Albopictus\;\text{presence})}= \frac{ \frac{[N \left( {C}_{ij} \right) -N(L\bigcap {C}_{ij})]}{N(L)} }{ \frac{[N(C)-N \left( L \right) -N \left( {C}_{ij} \right) +N(L\bigcap {C}_{ij})]}{[N(C)-N(L)]} } .\end{eqnarray*}

In Eq. (2) the numerator represents the proportion of *Ae. albopictus* presence pixels occurring in factor class *C*_*ij*_, while the denominator represents the proportion of *Ae. albopictus* absence pixels in factor class *C*_*ij*_. In Eq. (4) the numerator represents the proportion of *Ae. albopictus* absence pixels in factor class *C*_*ij*_, while the denominator represents the proportion of *Ae. albopictus* absence pixels in attributes excluding factor class *C*_*ij*_. The parameter *W*_*Cij*_ (*Ae. albopictus* presence) represents the weight of *C*_*ij*_ supporting the belief that *Ae. albopictus* presence exceeds *Ae. albopictus* absence. Parameter *W*_*Cij*_ (*Ae. albopictus* absence) is the weight of *C*_*ij*_ that supports the belief that *Ae. albopictus* absence exceeds presence.

After calculation of the EBF function for all *Ae. albopictus* presence conditioning factors, Dempster’s combination rule was introduced to produce the four integrated EBFs (Dempster, 1967). The formulae for combination of two *Ae. albopictus* presence conditioning factors _*C*1_ and _*C*2_ are as follows (Carranza, Woldai & Chikambwe, 2005):

(5) \begin{eqnarray*}& & {\mathrm{Bel}}_{{C}_{1}{C}_{2}}= \frac{{\mathrm{Bel}}_{{C}_{1}}{\mathrm{Bel}}_{{C}_{2}}+{\mathrm{Bel}}_{{C}_{1}}{\mathrm{Unc}}_{{C}_{2}}+{\mathrm{Bel}}_{{C}_{2}}{\mathrm{Unc}}_{{C}_{1}}}{1-{\mathrm{Bel}}_{{C}_{1}}{\mathrm{Dis}}_{{C}_{2}}-{\mathrm{Dis}}_{{C}_{1}}{\mathrm{Bel}}_{{C}_{2}}} \end{eqnarray*}

(6) \begin{eqnarray*}& & {\mathrm{Dis}}_{{C}_{1}{C}_{2}}= \frac{{\mathrm{Dis}}_{{C}_{1}}{\mathrm{Dis}}_{{C}_{2}}+{\mathrm{Dis}}_{{C}_{1}}{\mathrm{Unc}}_{{C}_{2}}+{\mathrm{Dis}}_{{C}_{2}}{\mathrm{Unc}}_{{C}_{1}}}{1-{\mathrm{Bel}}_{{C}_{1}}{\mathrm{Dis}}_{{C}_{2}}-{\mathrm{Dis}}_{{C}_{1}}{\mathrm{Bel}}_{{C}_{2}}} \end{eqnarray*}

(7) \begin{eqnarray*}& & {\mathrm{Dis}}_{{C}_{1}{C}_{2}}= \frac{{\mathrm{Dis}}_{{C}_{1}}{\mathrm{Dis}}_{{C}_{2}}+{\mathrm{Dis}}_{{C}_{1}}{\mathrm{Unc}}_{{C}_{2}}+{\mathrm{Dis}}_{{C}_{2}}{\mathrm{Unc}}_{{C}_{1}}}{1-{\mathrm{Bel}}_{{C}_{1}}{\mathrm{Dis}}_{{C}_{2}}-{\mathrm{Dis}}_{{C}_{1}}{\mathrm{Bel}}_{{C}_{2}}} .\end{eqnarray*}

Integrated EBFs of the *Ae. albopictus* presence conditioning factors are applied in sequence by means of Eqs. (5)–(7). Table 1 shows the estimated EBFs for the six *Ae. albopictus* presence conditioning factors.

**Table 1:** The estimated EBF for the six *Ae. albopictus* conditioning factors (i) altitude, (ii) slope, (iii) aspect, (iv) distance of locality from road, (v) distance of locality from river, and (vi) geology.

Layer	Classes	Pixels in class	Belief	Disbelief	uncertainty	plausibility
Altitude (m)	0–15	11844138	23	8	69	92
	15.01–27	11044489	24	8	68	92
	27.01–44	11103435	16	9	75	91
	44.01–70	11205803	12	9	79	91
	70.01–97	10777866	8	10	82	90
	97.01–127	10766882	2	10	88	90
	127.01–167	10729677	5	10	85	90
	167.01–228	10530594	0	11	89	89
	228.01–321	10384667	4	10	86	90
	321.01–2,031	10264963	1	10	89	90
Slope (Degree)	0–2.71	10817128	10	9	81	91
	2.72–5.12	11518857	12	9	79	91
	5.13–7.84	11211137	12	9	79	91
	7.85–10.55	11296609	10	10	80	90
	10.56–13.86	10691424	14	9	77	91
	13.87–17.48	10788198	13	9	78	91
	17.49–21.7	10695224	7	10	83	90
	21.71–26.82	10548139	5	10	85	90
	26.83–33.75	10586704	9	10	81	90
	33.76–76.85	10499094	4	10	86	90
Aspect (Direction)	Flat	1966236	8	11	81	89
	North	13716970	11	11	78	89
	Northeast	13146928	7	11	82	89
	East	12995205	6	11	83	89
	Southeast	13288273	10	11	79	89
	South	14219316	15	10	75	90
	Southwest	13358372	12	11	77	89
	west	12885860	13	10	77	90
	Northwest	13075354	13	10	77	90
Distance of locality from Road (m)	0–252.46	9093909	22	9	69	91
	252.47–757.38	15575127	19	8	73	92
	757.39–1,262.3	13129450	15	9	76	91
	1,262.31–1,767.22	11372444	9	10	81	90
	1,767.23–2,524.59	14065017	17	9	74	91
	2,524.6–3,281.97	10968822	4	10	86	90
	3,281.98–4,291.81	10815605	1	10	89	90
	4,291.82–5,554.11	8877260	3	10	87	90
	5,554.12–7,573.78	7524670	0	10	90	90
	7,573.79–64,124.67	7230210	6	10	84	90
Distance of locality from River (m)	0–9,601.28	9750778	11	9	80	91
	9,601.29–22,402.98	11907617	8	10	82	90
	22,402.99–35,204.69	11317607	8	10	82	90
	35,204.7–49,606.6	11872683	4	10	86	90
	49,606.61–65,608.73	11611532	14	9	77	91
	65,608.74–83,211.08	10954263	11	9	80	91
	83,211.09–102,413.63	10793879	6	10	84	90
	102,413.64–126,416.83	10642544	7	10	83	90
	126,416.84–161,621.51	10084369	16	9	75	91
	161,621.52–408,054.31	9717242	10	9	81	91
Geology	water	1483703	0	1	99	99
	clay or mud	17275519	5	1	94	99
	limestone	11355836	9	1	90	99
	delta	1287865	10	1	89	99
	alluvium	3199675	4	1	95	99
	sandstone	6712321	6	1	93	99
	beach sand	4633046	9	1	90	99
	sand	25077193	5	1	94	99
	dolostone (dolomite)	2012103	0	1	99	99
	mixed clastic/carbonate	27,891	0	1	99	99
	unconsolidated deposit	1691087	16	1	83	99
	calcarenite	958,785	14	1	85	99
	dune sand	71335	0	1	99	99
	silt	1399918	0	1	99	99
	indeterminate	537	0	1	99	99
	claystone	1138234	0	1	99	99
	terrace	363141	0	1	99	99
	carbonate	1479813	0	1	99	99
	shale	3770339	0	1	99	99
	mudstone	48266	0	1	99	99
	conglomerate	1250159	0	1	99	99
	black shale	21201	0	1	99	99
	greenstone	26169	0	1	99	99
	amphibolite	563162	0	1	99	99
	schist	960674	0	1	99	99
	mica schist	1854096	0	1	99	99
	quartzite	248931	0	1	99	99
	pyroxenite	12283	0	1	99	99
	phyllite	428634	0	1	99	99
	marble	20271	0	1	99	99
	felsic gneiss	276180	0	1	99	99
	tonalite	52635	0	1	99	99
	dacite	1823	0	1	99	99
	trondhjemite	7813	0	1	99	99
	slate	212254	0	1	99	99
	metasedimentary rock	1529772	4	1	95	99
	orthogneiss	87322	0	1	99	99
	granite	1855440	3	1	96	99
	quartz monzonite	32402	0	1	99	99
	granodiorite	39584	0	1	99	99
	granitic gneiss	2339458	2	1	97	99
	chert	1586586	4	1	95	99
Geology	quartz-feldspar schist	23548	0	1	99	99
	mafic gneiss	146884	0	1	99	99
	mylonite	158219	0	1	99	99
	biotite gneiss	3561030	1	1	98	99
	gneiss	1525144	0	1	99	99
	gabbro	148560	0	1	99	99
	ultramafic intrusive rock	13,298	0	1	99	99
	amphibole schist	11552	0	1	99	99
	hornfels	3420	0	1	99	99
	charnockite	8768	0	1	99	99
	augen gneiss	19131	0	1	99	99
	quartz diorite	77694	0	1	99	99
	arkose	123	0	1	99	99
	gravel	120,094	0	1	99	99
	loess	92	0	1	99	99
	tectonic breccia	479	0	1	99	99
	biotite schist	17160	0	1	99	99
	metamorphic rock	1063128	0	1	99	99
	siltstone	66319	0	1	99	99
	graywacke	153019	0	1	99	99
	diorite	15751	0	1	99	99
	peat	449404	0	1	99	99
	metavolcanic rock	529438	0	1	99	99
	felsic metavolcanic rock	928277	0	1	99	99
	mafic metavolcanic rock	138759	0	1	99	99
	syenite	5883	0	1	99	99
	paragneiss	72466	0	1	99	99
	lake or marine deposit (non-glacial)	1159949	0	1	99	99
	granitoid	136910	0	1	99	99
	phyllonite	15496	0	1	99	99
	arenite	28543	0	1	99	99
	meta-argillite	570737	0	1	99	99
	intermediate metavolcanic rock	46,641	0	1	99	99
	migmatite	33447	0	1	99	99
	diabase	6843	0	1	99	99
	norite	14	0	1	99	99
	felsic volcanic rock	38	0	1	99	99
	pelitic schist	13	0	1	99	99
Geology	meta-conglomerate	1160	0	1	99	99

### Climatic data, future scenarios and climate models

Baseline climate was represented by the WorldClim current climate dataset of BIOCLIM variables (http://www.worldclim.org). WorldClim is a high-resolution climate average for the period 1961 to 1990, with global coverage and spanning the time period over which the majority of occurrence records were collected. Possible future climates at global scale incorporated four IPCC5 greenhouse gas concentration (GHC) trajectories, which differ in terms of GHC emission peaks. The lower the number of the trajectory, the earlier in the century it peaks.

We purposefully chose the worst (extreme) RCP8.5 (peak 2080) (Stocker et al., 2013) for incorporation into the future climate scenario in the model projections as it is not yet possible to determine which estimates of the climate change and RCPs of 2.6, 4.5, 6.0 and 8.5 are the most reliable (Randall et al., 2007). RCP8.5 is a representative concentration pathway that includes relatively high emissions of greenhouse gases. Other factors assumed in RCP8.5 are high demographic development, relatively slow economic growth, with modest progress in technology and the introduction of novel sources of energy. These factors culminate in an increased demand for energy and higher GHG emissions over the long term, without a more radical approach to the projected impact of climate change (Riahi et al., 2011).

There are 19 General Circulation Models (GCMs) in WorldClim database and we have selected GCMs of *Miroc3.2* and *CSIRO-MK30,* which have higher reputations and have been used for projections of many invasive species, agricultural crops and pests (Da Silva et al., 2017a; Da Silva et al., 2017b; Lamsal et al., 2017; Paterson et al., 2017; Ramirez-Cabral, Kumar & Shabani, 2017b; Ramirez-Cabral, Kumar & Shabani, 2017a; Shabani, Kumar & Ahmadi, 2016; Shabani, Kumar & Ahmadi, 2017; Shabani, Kumar & Taylor, 2012).

### Climatic modeling

MaxEnt desktop version 3.3.3k, with modified parameters, was used to construct the climatic model (Phillips, Anderson & Schapire, 2006; Phillips & Dudík, 2008). MaxEnt requires a user-defined background of geographical data (Guillera-Arroita, Lahoz-Monfort & Elith, 2014) in order to compare the climate of a set of grid cells representing the presence of a species, with the reference set representing the climate of the sampled cells. The selected geopgraphical data is a significant determinant of the results of the model (Elith et al., 2011) and it is important that it reflect all environmental variations covering the areas representing the presence of the species (Elith, Kearney & Phillips, 2010). The algorithm in MaxEnt estimates the maximum entropy probability distribution that approximates uniformity, based on a comparison of presence and background location interactions with a set of variables, limited by parameters imposed by the observed spatial distributions and environmental factors. Optimisation of the maximum entropy probability distribution is achieved by minimisation of the relative entropy between presence and background point data (Phillips, Anderson & Schapire, 2006). MaxEnt, with inbuilt MESS analysis tool, has the capacity to predict future distributions, generated from two datasets of environmental variables (Elith et al., 2011). In our study, the current conditions are used to generate the model, with a set of variables utilized for projection of the future scenario (in this case 2055).

Using jackknife analysis and Pearson correlation technique to correlate coefficients, we selected the most influential variables showing low correlation (*R*^2^ < 0.5) for this modeling study. Here, BIO11 (Mean Temperature of Coldest Quarter), BIO16 (Precipitation of Wettest Quarter) and BIO17 (Precipitation of Driest Quarter) were selected for the modeling. To achieve greater consistency of background data and overcome the potential for finding fewer records representing areas more recently experiencing invasions, as well as those incompletely sampled, we assigned greater prominence to the records representing less geographical proximity. However, it should be noted that without information on actual survey returns, there is no method of separating unsuitable and under-sampled areas, and that the weighting of prominence cannot overcome the fusion of these two categories of data. After using the Gaussian kernel method to establish deviations from the ArcGIS default values, the formula applied for weighting is to divide total weighted records by the weighted number of land cells of the specific area, to exclude coastal region edge effects. By adjusting the resulting grid to a range of 1–20, extreme values were excluded. This weighting method, as advocated by Elith, Kearney & Phillips (2010), reduces bias that gives prominence to the records of more densely sampled areas. Background training points were generated from the kernel density layer for the species using Hawths Tools extension (Beyer, 2004).

### Model validation

The non-climatic modeling analysis was executed and validated using known *Ae. albopictus* presence (Fig. 1). Using training and testing *Ae. albopictus* presence data, validation was carried out using the area under curve (AUC) method. While training presence data was used to generate the model, the results using this data does not fully represent the model’s total efficiency. The prediction rate was measured to establish how efficiently the model and selected conditioning factors predicted *Ae. albopictus* presence. AUC can assess prediction accuracy qualitatively by arrangement of the calculated values of all cells of the study locations into descending order, providing an individual hierarchical ranking of the accuracy of each prediction. Thereafter, the values of cells were divided into 100 classes with accumulation intervals of 1%.

## Results

### Non-climatic modeling

We examined, and assessed individually, six geological variable factors that directly impact the presence of *Ae. albopictus* in a specific locality. The altitude EBFs indicated that localities of 0 to 97 m above sea level had a high probability presence of *Ae. albopictus*. The belief value (*Bel*) peaked at 24 with altitudes from 15 to 27 m, while it was 5 and 0 at altitudes from 127 to 167 m and 167 to 228 m respectively (Table 1). Slope EBF indicated that classes of 2.72 to 7.84°, 10.56 to 13.86°and 33.76 to 76.85°produced *Bel* values of 12, 14 and 4, respectively. The three highest Aspect *Bel* values of 15, 13 and 12 related to the classes of South, Northwest and Southwest, respectively (Table 1). Distance from locality to road was included as a conditioning factor, as motor vehicles have been shown as a means of *Ae. albopictus* transmission. The highest *Bel* values for this factor were 22 and 19, representing the classes of 0 to 252 m and 252 to 757 m, respectively. For Distance of locality from river, EBF estimated the probability of *Ae. albopictus* presence, for all ten classes (Table 1). For Geology, the classes of unconsolidated deposit, calcarenite and delta scored *Bel* values of 16 and 14 and 41, indicating the probability of *Ae. albopictus* presence in these geological formations (Table 1).

**Figure 4:**
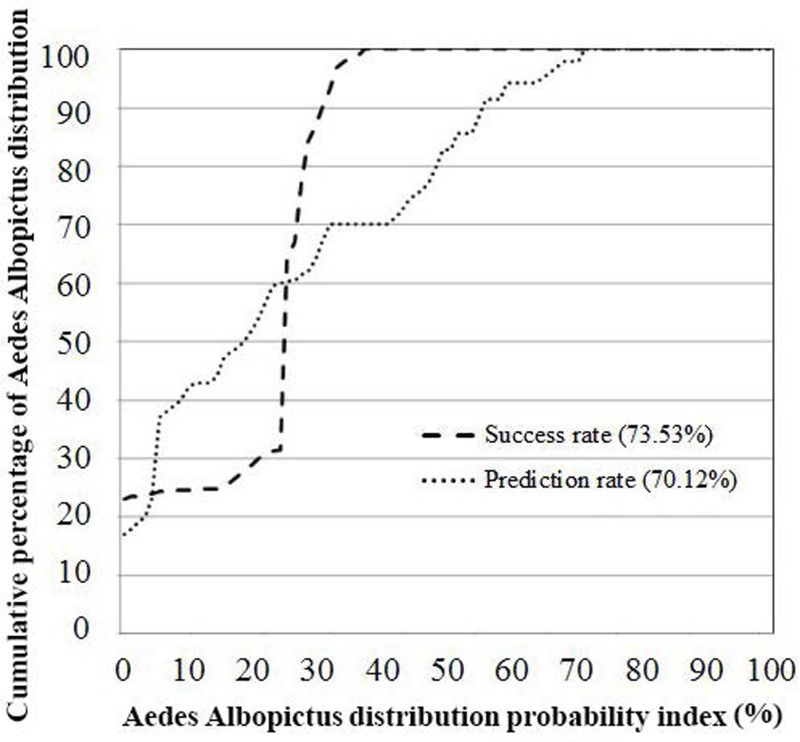
AUC- success rate and prediction rate of EBF method.

**Figure 5:**
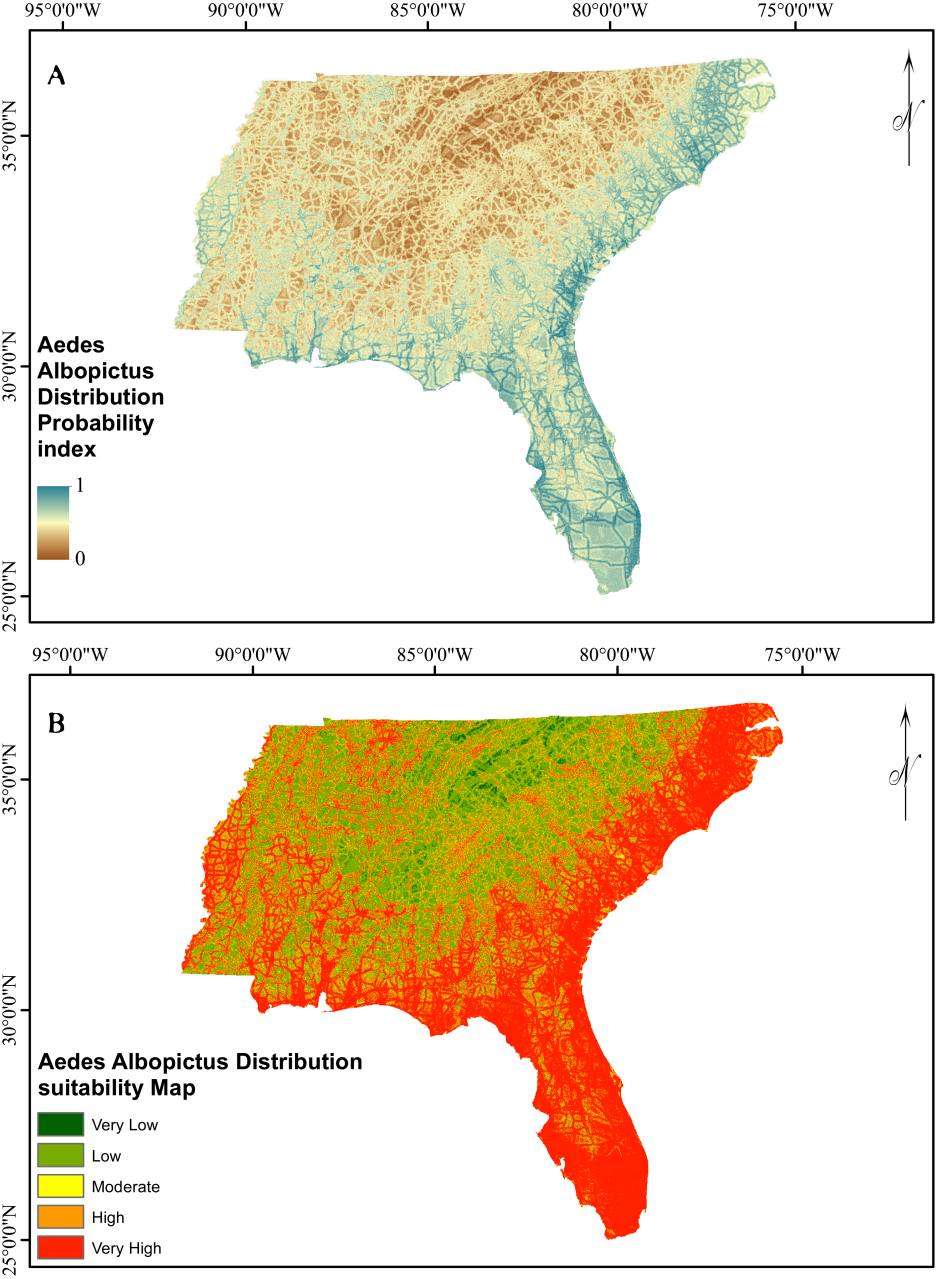
Asian Tiger Mosquito probability and susceptibility maps achieved by EBF method.

### Probability index and suitability map

The verification results for EBF model are shown in Fig. 4. Probability index maps of *Ae. albopictus* presence produced by EBF method are shown in Figs. 5A and 5B respectively. The range is from 0 to 1, where 0 represents zero probability and 1 represents 100% probability. For producing suitability maps, as well as improving the visual interpretation of locational suitability, probability maps require some form of classification (Umar et al., 2014). There are a variety of classification techniques such as equal interval, natural break, standard deviation and quantile, the selection of which should be based on the research data characteristics and study objectives. Equal interval is suitable when the data displays a normal distribution, while standard deviation arranges the data into a fixed number of classes. Natural break suits a dataset exhibiting a sudden or big jump. Here, in order to have a reliable judgment regarding the impact of every class of each factor on species occurrence, we attempted to reduce the influence of classification algorithm on the conditioning factors classes as much as possible. In some population analysis projects where the goal is to find a big jump in the data, natural break technique is highly recommended (Hui et al., 2010; Umar et al., 2014), while in this research, this method would not be efficient. Hence, quantile-based classification technique was found to be more appropriate to classify the factors in this study. This method groups equal number of pixels (area) into each group without any interference in the separation of the pixels. We thus selected the quantile method to produce the suitability classes. The verification results for EBF model are shown in Fig. 4. The AUC results showed 0.73 success rate and 0.70 prediction rate (Fig. 4) and these values are high enough and satisfactory for model prediction as documented in Umar et al. (2014). Our probability indexes were into five zones of suitability: very low, low, moderate, high, and very high, for EBF output (Fig. 5B).

### Climatic modeling

**Figure 6:**
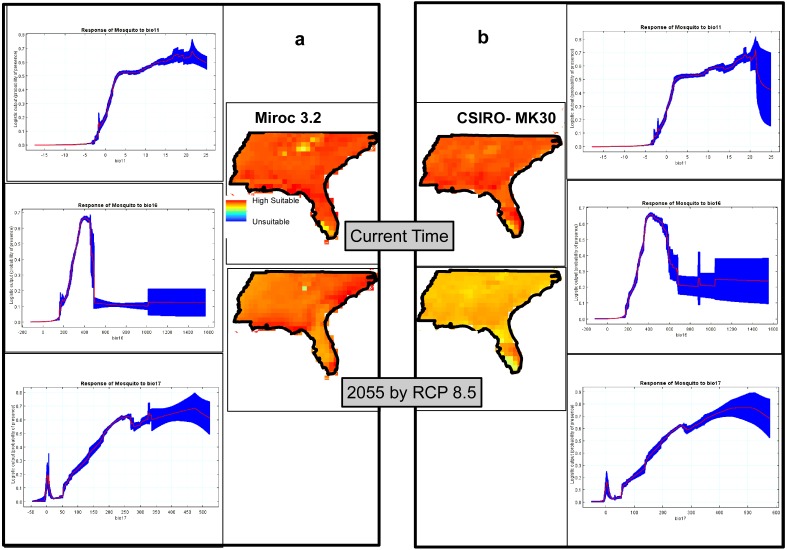
Climatic suitability maps of Asian Tiger Mosquito based on two General Circulation Models of (A) Miroc3.2 and (B) CSIRO-MK30 under RCP 8.5 scenario through MaxEnt software plus the response curves of the most important climatic layers.

The climatic model produced by MaxEnt, using two GCMs, *Miroc3.2* (Fig. 6A) and *CSIRO-MK30* (Fig. 6B), under the RCP 8.5 scenario, shows virtually the whole study site is highly suitable for *Ae. albopictus* and that this condition will persist until at least 2055. Comparing the GCM projections for 2055, *CSIRO-MK30* produced a more moderate pattern of climatic suitability than *Miroc3.2*. Both GCM response curves show the highest probabilities of *Ae. albopictus* presence in areas with Coldest Quarter Mean Temp (bio11) from 16 to 23 °C, Wettest Quarter Precipitation (bio16) of 430 mm and Driest Quarter Precipitation (bio17) of 350 to 450 mm (Figs. 6A and 6B). The *Miroc3.2* mean AUC was 0.868, while *CSIRO-MK30* indicated 0.864.

## Discussion

This study undertook a comparative assessment of the proficiency of the EBF and MaxEnt statistical methods in mapping the probability of *Ae. albopictus* expansion based on climatic and non-climatic parameters respectively. Based on AUC validation method, both EBF and MaxEnt had high prediction rates and thus both can be used to generate *Ae. albopictus* expansion probability and suitability for current and future time. Such maps would assist national, regional and local public health organizations in the identification of areas, and their degree of suitability to *Ae. albopictus* expansion or invasion, as a blueprint on which to plan and implement prevention or reduction measures, or to prepare for potential invasion. Suitability maps provide a foundation for more refined analytical tools such as hazard and risk mapping. It is important to note that the accuracy of *Ae. albopictus* expansion risk is dependent on the accuracy with which the conditioning factor values are calculated. Beyond the establishment of the class, it is essential to understand which conditioning factors impact most on *Ae. albopictus* expansion or invasion. Once the conditioning factors and associated severity of impact have been established, the information is valuable as a foundation to conservation strategies to protect areas at risk.

We also highlight that through the *Miroc 3.2* model, the overall suitability remains the same by 2055, while the suitability will slightly decrease by 2055 in the *CSIRO-MK30* model and the possible explanation of this difference is due to each GCM and SDM functioning slightly differently and, in line with this matter, Shabani, Kumar & Ahmadi (2017) has recently documented that comparison of the individual SDM or GCM to an ensemble approach showed that there was a better agreement between the ensemble outputs under different GCMs or SDMs. This finding is in line with Araújo & New (2007), who have recommended that using ensemble forecasting has clear advantages over single model forecasts.

Our results indicate the importance of both climate and non-climate factors on the degree of potential *Ae. albopictus* expansion. Complementary to this finding, a number of studies have shown the inability of diapausing *Ae. albopictus* eggs to survive extreme winter temperatures (Hanson & Craig, 1994). Urban habitats with high levels of organic material, such as sewerage treatment works and storm water drainage systems, can impact on the extent of *Ae. albopictus* expansion and such larval habitats should be treated with well-developed methods providing long term relief for the entire *Ae albopictus* season (Rochlin et al., 2013b). Our results also indicate that in terms of climatic suitability, and predicted future climate scenarios, this study has validity and will remain valid in the future for *Ae. albopictus* (Fig. 6), particularly for the USA. Almost one-third of the study site was identified as being at high risk of *Ae. albopictus* expansion, based on the location of suitable non-climatic parameters alone (Fig. 5). Our results show the importance of non-climatic parameters in that these can be used to further refine high probability areas within climatically suitable regions. Thus, in terms of offering *Ae. albopictus* control services, the climatic result is not as useful on a practical basis as the non-climatic result due to the overall climate suitability of the whole study site. However, the projected future impact of non-climatic parameters on *Ae. albopictus* expansion for the future was not undertaken as the road and river layers will change.

The EBF outputs for altitude conditioning factor indicated that areas from 0 to 97m above sea level had a high probability of *Ae. albopictus* presence, which may be attributable to the greater instability of organic material, water or other non-climatic factors at higher elevations. Moisture preservation and distribution of vegetation amount are related to slope and aspect. Results showed that these factors impacted specifically on the initiation of expansion, as well as having a direct impact on suitability to expansion. The EBF outputs on distance of locality from road and river indicated that both factors had significance in *Ae. albopictus* expansion, which may be due to the greater transportability of *Ae. albopictus* eggs by vehicles, on rivers and in water catchment areas. Conversely, it is probable that geology does not impact significantly on *Ae. albopictus* expansion. Thus, altitude, slope, aspect, distance of locality from road, and distance of locality from river are the most significant non-climatic factors affecting expansions of *Ae. albopictus*.

Community education regarding *Ae. albopictus* and awareness campaigns as to home and garden sanitation and interventions from all levels of public, environmental health and vector control units, as well as private sector infestation control offering mosquito control to provide barrier treatments or other specific locality eradication methods is important. The efficiency and practicality of large-scale adulticiding should be researched, as well as determining the combination of factors which would demand the initiation of this control. Without ongoing strategies to prevent *Ae. albopictus* further expansion, the problem will have to be faced on an increased scale in the near future. Ongoing research has been examining controls involving genetic modification of the species, as well as RIDL (release of insects with dominant lethality) and the introduction of *Wolbachia* bacterium, an insect parasite (Walker, 2016).

## Conclusion

Projected warmer winter temperatures, increasing gradually over the next few decades, will impact significantly on the potential for greater *Ae. albopictus* expansion of range in the southeastern and eastern USA. By implication, more people will live within *Ae. albopictus* range, and will potentially be subjected to more bites from the greater density of the species, thus being at greater risk of the posed arboviral threats of the species. At present, aside from small scale direct extermination of hatchings and prophylactic restriction of the specified semi-enclosed moist habitats of water and organic matter containers, by minimizing such habitats, no strategies or techniques of larger area control exists. Thus, public health agencies, particularly in regions with little or no broad anti-mosquito strategies and techniques, may find themselves in a vacuum, in terms of vector potential of *Ae. albopictus* and a novel pathogen.

Statistical modeling is advantageous for its simplicity and user friendly qualities throughout the suitability mapping process involved. It is also capable of processing large quantities of case or region-specific GIS data relatively quickly. Sustainable urban development is dependent on effective remedies to the potential health impacts of vector hazards that can reach epidemic proportions. Our study indexed potential non-climatic factors and delineated high risk regions, demonstrating an investigative and analytical approach as a foundation for the policy makers and public health networks. We reiterate that anticipating areas of potential establishment based on non-climatic factors is the priority practical approach, where a whole region is classified as suitable for *Ae. albopictus* range extension.

##  Supplemental Information

10.7717/peerj.4474/supp-1 Supplemental Information 1 Inventory Details of Inventory classification.

10.7717/peerj.4474/supp-2 Supplemental Information 2 Masked layers Masked layers used for this study.

## Additional Information and Declarations

### Data Availability

The following information was supplied regarding data availability: The raw data is provided in the Supplemental Files.
